# Serum total IgE, ascaris lumbricoides specific IgE and eosinophils in parasites-infected children in a tropical area

**DOI:** 10.1186/1939-4551-8-S1-A223

**Published:** 2015-04-08

**Authors:** Valéria Soraya De Farias Sales, Edna Marques Araújo Silva, Sarah Dantas Viana Medeiros, Luanda Bárbara Ferreira Canário Souza, Vanessa Marques Araújo Silva, Tereza Neuma De Souza Brito, Paula Renata Lima Machado

**Affiliations:** 1 Federal University of Rio Grande Do Norte, Brazil

## Background

This study investigated the relationship between total IgE and *Ascaris lumbricoides* specific IgE and eosinophils in children from endemic areas to assess the Th2-type immune response in a population that comprised 205 children with aged 1-10 years, and of low socioeconomic status.

## Methods

Fecal samples were analysed by the methods of Blagg and Kato-katz. The serum levels of total IgE and *A. lumbricoides* specific IgE were determinated by ImmunoCAP (Pharmacia) and the eosinophils were counted in peripheral blood.

## Results

The results showed a prevalence of 89% (182) for intestinal parasites. *A. lumbricoides* was detected in 140 (68%) children. The levels of total and specific IgE and eosinophils presented values above those of standard reference (median 480 KU/L and 0,74 KUA/L and 8%, respectively). Total IgE, *A. lumbricoides* specific IgE and eosinophils were significantly higher in the *A. lumbricoides* positive children as compared to *A. lumbricoides* negative ones (p = 0.02, <0.01 and 0.03).

## Conclusions

Our results showed that parasites intestinal infection, particularly *A. lumbricoides*, induced a Th2-type immune response with production of the total and specific IgE and eosinophils.

